# Supplementary material for: Diverse Localization Patterns of an R-Type Lectin in Marine Annelids
Source: Molecules. 2021 Aug 7;26(16):4799. doi: 10.3390/molecules26164799 (PMC8399747; doi:10.3390/molecules26164799)
Supplement: Supplementary file 1 [file molecules-26-04799-s001.zip › molecules-1289447-supplementary.pdf]

Supplemental Figures

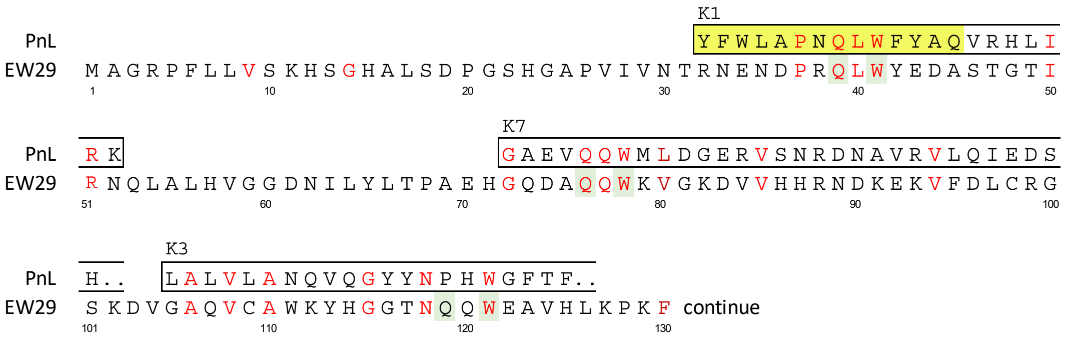

Suppl. Figure S1. Antibody design of PnL. The upper sequence is the partial sequence derived from the lysyl-endopeptidase-cleaved polypeptides of PnL [5]. The lower sequence is the N-terminal sequence (1–130) of the earthworm lectin EW29. The number of K corresponds to the peptides in a previous report [5]. The yellow ribbon in PnL is the part of the 14-amino-acid sequence used to immunize the rabbits. The olive highlights in EW29 are the Q-X-W sequences characteristic of the R-type lectin family. The red amino acids are identical in bothR-type lectins.

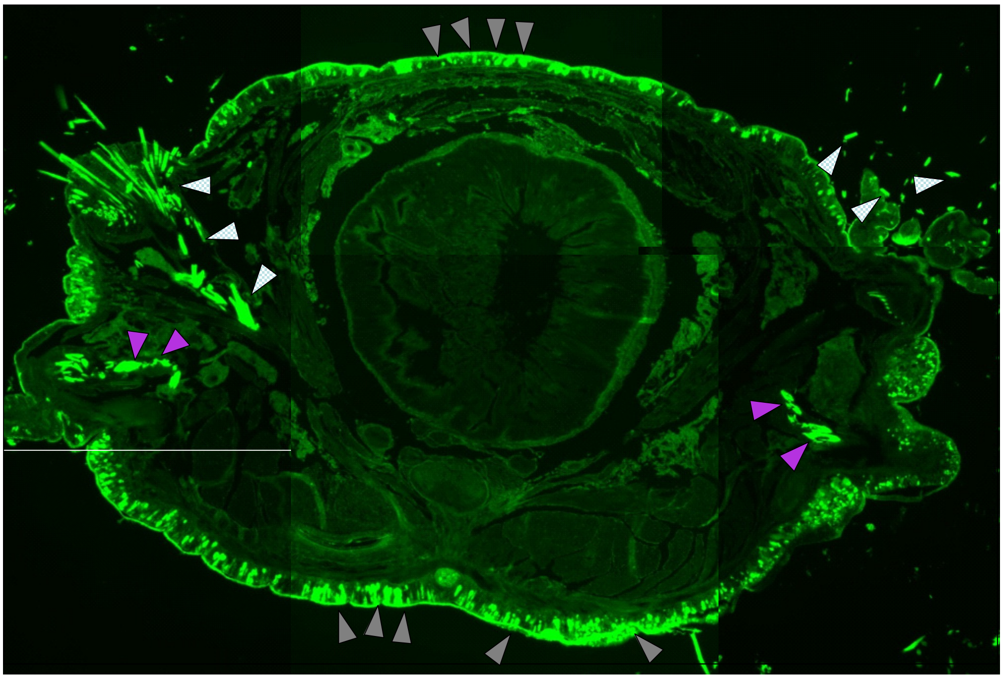

Suppl. Figure S2. The binding pattern of FITC-labeled *Agaricus bisporus* agglutinin (ABA) on the cross-section of the lugworm tissue. Triangles indicate the tissues and organs of the epidermis (gray), acicula and setae (meshed white), and nephridium (purple) responding to the figure 3B. This figure was updated the previously published data [11].
